# Exploring the Impact of Polychlorinated Biphenyls (PCBs) on the Development of MASLD: A Comprehensive Review

**DOI:** 10.3390/cells15040364

**Published:** 2026-02-18

**Authors:** Valeria Longo, Giuseppa Augello, Noemi Aloi, Alessandra Cusimano, Anna Licata, Emanuele Cannizzaro, Melchiorre Cervello, Maurizio Soresi, Paolo Colombo, Lydia Giannitrapani

**Affiliations:** 1 Institute for Biomedical Research and Innovation (IRIB), National Research Council, 90146 Palermo, Italy; valeria.longo@irib.cnr.it (V.L.); giuseppa.augello@irib.cnr.it (G.A.); noemi.aloi@irib.cnr.it (N.A.); alessandra.cusimano01@unipa.it (A.C.); melchiorre.cervello@irib.cnr.it (M.C.); 2 Department of Biological Chemical and Pharmaceutical Science and Technology (STEBICEF), University of Palermo, 90128 Palermo, Italy; 3 Department of Health Promotion Sciences, Maternal and Infant Care, Internal Medicine and Medical Specialties (PROMISE), University of Palermo, 90127 Palermo, Italy; anna.licata@unipa.it (A.L.); emanuele.cannizzaro@unipa.it (E.C.); maurizio.soresi@unipa.it (M.S.)

**Keywords:** polychlorinated biphenyls, metabolic dysfunction-associated steatotic liver disease, non-alcoholic fatty liver disease, toxicant-associated steatotic liver disease, dioxin-like polychlorinated biphenyls, non-dioxin-like polychlorinated biphenyls

## Abstract

**Highlights:**

Experimental, epidemiological, and mechanistic evidence links polychlorinated bi-phenyl (PCB) exposure to liver injury, steatosis/steatohepatitis, fibrosis, and hepato-carcinogenesis, with a focus on congener profiles and susceptibility factors (e.g., sex, metabolic comorbidities). PCB-driven molecular pathways in hepatocytes and hepatic non-parenchymal cells, specifically in aryl hydrocarbon receptor (AhR), constitutive androstane receptor (CAR), and pregnane X receptor (PXR) signaling, oxidative stress, mitochondrial dysfunction, lipid metabolism reprogramming, inflammatory/immune responses, have implications for liver disease progression.

**What are the main findings?**

- Chronic PCB exposure is consistently associated with liver dysfunction and MASLD phenotypes in both humans and experimental models.
- PCBs disrupt hepatic homeostasis by converging on a limited set of pathways (AhR, CAR/PXR), inducing oxidative and ER stress, mitochondrial impairment, and the dysregulation of lipid and glucose metabolism.

**What are the implications of the main findings?**

- There is an impact on public health and clinical practice.
- We suggestcandidate biomarkers and therapeutic targets.

**Abstract:**

Metabolic dysfunction-associated steatotic liver disease (MASLD), formerly known as non-alcoholic fatty liver disease (NAFLD), is becoming the most common liver disease, affecting between 30 and 40% of the global population. MASLD is a multifaceted disease spectrum that is closely associated with obesity, insulin resistance, type 2 diabetes mellitus and, more broadly, metabolic syndrome. All these conditions increase the risk of liver-related mortality, which explains the intense research efforts in recent years to better elucidate its pathogenesis. The crucial impact of environmental pollutants on the development of MASLD is now well recognized. Polychlorinated biphenyls (PCBs) are environmental contaminants that act as endocrine disruptors. Recently, they have been associated with the development of diabetes, obesity, MASLD, and cancer. The association between liver diseases, namely toxicant-associated steatotic liver disease and steatohepatitis (TASLD and TASH, respectively), and occupational exposure to PCBs and other industrial chemicals has been documented by several lines of evidence, whereas the potential role of low-level environmental pollution in liver disease and in MASLD remains incompletely understood. Previous studies on animal models have shown that PCB exposure is associated with steatosis/steatohepatitis, fibrosis, cirrhosis, hepatocellular carcinoma (HCC), altered liver enzymes, and mortality in exposed populations. This review investigates the mechanisms underlying hepatic steatogenesis in preclinical and animal models and analyzes the existing literature on the possible role of PCBs, together with the other conventional risk factors, in the development of MASLD in humans.

## 1. Introduction

Exposure to environmental pollutants such as heavy metals, persistent organic pollutants (POPs), pesticides, and industrial chemicals has been increasingly recognized as a risk factor for liver disease. These substances can accumulate in the liver, the body’s main detoxification organ, where they interfere with metabolic processes and promote cellular stress. Over time, chronic exposure has been associated with a higher risk of developing conditions such as metabolic dysfunction-associated steatotic liver disease (MASLD; formerly non-alcoholic fatty liver disease, NAFLD), fibrosis, cirrhosis, and even liver cancer, highlighting the liver’s vulnerability to environmental toxicants and the importance of reducing the pollutant exposure for liver health [1].

The difference between toxicant-associated steatotic liver disease (TASLD) and MASLD lies in their etiologies and risk factors. In both cases, pathogenesis involves lipotoxicity, mitochondrial dysfunction, and chronic low-grade inflammation, leading to hepatocyte injury, lobular inflammation, and fibrosis. MASLD is highly associated with metabolic comorbidities and is now a leading cause of chronic liver disease worldwide [2,3,4]. However, given the rapid spread of risk factors for both MASLD and TASLD in the general population, especially in Western countries, it is sometimes difficult to keep the two entities separate.

Polychlorinated biphenyls (PCBs) are a group of synthetic organic chemicals once widely used in industrial applications due to their chemical stability, non-flammability, and insulating properties. Produced commercially from the 1930s until their ban in many countries in the late 1970s, PCBs were employed in electrical equipment, hydraulic fluids, and as plasticizers. PCBs are persistent lipophilic pollutants that bioaccumulate in humans in adipose tissues and concentrate in the liver, a major site of biotransformation and lipid storage. PCBs comprise 209 congeners, classified based on the number and position of chlorine atoms on the biphenyl rings. They are broadly divided into non-dioxin-like PCBs (NDL-PCBs) and dioxin-like PCBs (DL-PCBs), which differ in their chemical structure, receptor targets, and toxicological profile (Table 1).

Among these PCB families, the most common DL-PCBs are PCB-126, -169, -77, and -81 (non-ortho; the most potent) and PCB-105, -114, -118, -123, -156, -157, -167, and -189 (mono-ortho; lower potency). DL-PCBs share structural and functional similarities with polychlorinated dibenzo-p-dioxins (PCDDs) and polychlorinated dibenzofurans (PCDFs). They are planar congeners whose mixture potency is commonly normalized using toxic equivalents (TEQs), summing each congener concentration × its toxic equivalency factor (TEF) relative to TCDD (2,3,7,8-tetrachlorodibenzo-p-dioxin). This approach enables cross-study comparisons of aryl hydrocarbon receptor (AhR)-mediated activity, asAhR is a ligand-activated transcription factor that regulates the expression of multiple xenobiotic-responsive genes. In contrast, NDL-PCBs (e.g., PCB-28, -52, -101, -138, -153, -180) are ortho-substituted non-planar congeners that largely do not act via AhR and, therefore, are not assigned TEFs. In this case, the risk is typically calculated by congener sums (e.g., ICES-6, as defined by the Standing Committee on the Food Chain and Animal Health) rather than TEQs. This dichotomy has crucial implications: TEQ captures only the DL fraction of PCB risk, but human body burdens are dominated by NDL congeners [5,6]. NDL-PCBs tend to dominate in human matrices (serum/adipose tissue), whereas DL-PCBs contribute disproportionately to toxic potency (via TEQ).

Increasing evidence suggests that PCB exposure may contribute, as an additional risk factor, to the onset and progression of MASLD, the most common chronic liver disease worldwide, affecting ~30–40% of adults and with a prevalence projected to rise further, meaning that even modest toxicant effects can translate into a large population burden [3,7].

This review summarizes the most recent literature data on these newly recognized environmental risk factors for MASLD in order to investigate the molecular mechanisms underlying hepatic steatogenesis.

## 2. Liver Steatosis: Old and New Nosographic References

Liver steatosis, as first described by Ludwig et al. [8] in 1980, was a condition understood to be mimicking alcoholic hepatitis in people who did not consume a significant amount of alcohol. Today, under its new definition as MASLD, it has been identified as a key component of the metabolic dysfunctions typical of metabolic syndrome (type 2 diabetes mellitus—T2DM, arterial hypertension, visceral obesity, hypertriglyceridemia, and low high-density lipoprotein levels) [9]. However, this condition is not particularly rare in non-obese non-diabetic adults, i.e., in patients with BMI <25 and normal metabolic profiles, a condition known as “lean MASLD” [9,10].

As documented by the literature data, TASLD and its evolved form TASH (toxicant-associated steatohepatitis) are caused by exposure to hepatotoxic environmental or occupational chemicals—such as PCBs, vinyl chloride, or other industrial toxicants—in individuals who do not have traditional metabolic risk factors like obesity, diabetes, or significant alcohol consumption. Its pathogenesis is driven by direct chemical injury, oxidative stress, and inflammatory signaling triggered by toxicant exposure, resulting in hepatic steatosis, inflammation, and sometimes fibrosis and cancer. TASLD can occur in lean individuals and may be characterized by normal liver enzymes, making diagnosis challenging. In this situation, conventional metabolic risk factors are typically absent, and the disease is linked to specific chemical exposures [11,12].

Conversely, MASH (metabolic dysfunction-associated steatohepatitis) is the progressive inflammatory form of MASLD, formerly known as non-alcoholic steatohepatitis (NASH), which represented the advanced stage of NAFLD, which was the previous terminology used to describe the spectrum of liver diseases characterized by liver steatosis in individuals not consuming a significant amount of alcohol. The most relevant difference between the two definitions is that MASLD requires the presence of steatosis and at least one cardiometabolic risk factor (CMRF)—such as obesity, T2DM, dyslipidemia, or hypertension—whereas NAFLD is defined primarily by the exclusion of significant alcohol intake and other causes, without a mandatory metabolic criterion [13]. MASLD and MASH are the updated terms thatemphasize how metabolic dysfunction is central to the pathogenesis and clinical consequences of the disease.

Historically, PCBs have been strongly implicated in the pathogenesis of TASH, resulting from exposure to industrial chemicals rather than metabolic syndrome or alcohol. Both animal and human studies demonstrate that chronic PCB exposure, particularly to DL-PCBs like PCB-126 and PCB-56, induces hepatic steatosis, inflammation, hepatocyte necrosis, and fibrosis, closely mirroring the histopathology of MASH [14,15].

More recently, PCBs as well as organochlorine pesticides, heavy metals, and micro/nanoplastics, have been increasingly recognized as contributors to MASLD pathogenesis, promoting hepatic lipotoxicity, mitochondrial dysfunction, oxidative inflammation, and gut dysbiosis, thereby exacerbating steatosis and its progression to steatohepatitis and fibrosis [16]. In addition, the Guidelines from the American Association for the Study of Liver Diseases (AASLD) and recent reviews have emphasized that environmental exposures—especially during critical windows such as early life and adolescence—interact with genetic, dietary, and metabolic factors to accelerate MASLD onset and progression, supporting the “multiple-hits” model of disease pathogenesis [17].

Moreover, epidemiological studies and mechanistic data demonstrate that chronic low-dose exposure to these agents is associated with increased MASLD risk and severity, with additive and synergistic effects being observed when multiple toxicants are present [16]. This model integrates environmental toxicants as amplifiers of hepatic injury in the context of pre-existing metabolic risk, underscoring the need for comprehensive risk assessment and mitigation strategies in clinical practice.

## 3. PCB Entry Routes into the Body

For the general population, the predominant route of exposure to PCBs is dietary ingestion. Due to their lipophilic and persistent nature, PCBs bioaccumulate in the food chain, with fatty fish, meat, dairy products, and other animal-derived foods representing the main sources [18]. Additional contributions may come from contaminated drinking water and incidental ingestion of soil or dust, particularly in children [19]. Following ingestion, the intestinal absorption is highly efficient (>90% for many congeners), with subsequent distribution primarily to the liver and adipose tissue [20].

Inhalation constitutes a relevant exposure pathway, particularly for semi-volatile lower-chlorinated congeners. Indoor sources include PCB-containing caulking, paint, and fluorescent light ballast in older buildings, while outdoor exposure may occur in areas near waste incineration or contaminated sites. In urban and indoor environments, inhalation exposure can be comparable to dietary intake. After inhalation, PCBs rapidly cross the alveolar barrier and are distributed to lipid-rich tissues [20,21,22,23].

Dermal absorption is a less common but occupationally-relevant pathway. Direct contact with PCB-containing oils, soils, or sediments may lead to systemic uptake, with lipophilic congeners penetrating the skin and accumulating in fat and liver tissue [21].

Finally, transplacental transfer and lactational exposure are critical for early-life exposure. PCBs cross the placenta and accumulate in fetal tissues, with levels often reflecting maternal serum and cord blood concentrations. Postnatally, breast milk constitutes a major source of exposure as PCBs preferentially partition into milk fat. These exposures are of particular concern given their occurrence during sensitive developmental periods [24].

Both DL-PCBs and NDL-PCBs contribute to steatosis, but they do so through distinct and often complementary mechanisms. Understanding this dichotomy is crucial for comprehending the full toxicological profile of PCB mixtures. In the following sections, the specific biological mechanisms induced by the two families of PCBs are analyzed.

## 4. Human Studies

Human epidemiology provides consistent data linking PCB body burden to biochemical and clinical indicators of liver injury. In U.S. adults (NHANES 2003–2004), higher lipid-adjusted PCB levels were associated with unexplained alanine aminotransferase (ALT) elevation, a proxy for suspected fatty liver, after adjustment for metabolic covariates [7].

Multiple cohort studies from different countries have shown that serum PCB concentrations, which are related to PCB exposure, are significantly associated with more severe hepatic involvement as well as insulin resistance, dyslipidemia, and increased proinflammatory cytokines, supporting the role of PCBs as endocrine and metabolic disruptors in MASLD pathogenesis [25].

Similarly, a population study from Anniston, Alabama, found higher alanine aminotransferase (ALT)/aspartate aminotransferase (AST)/gamma-glutamyl transferase (GGT) levels with higher PCB exposures. Extending our analysis to northern Europe, a Finnish general population cohort associated serum POPs, including PCBs, with liver disease incidence and biomarker abnormalities. Recent analyses from China (*n* ≈ 1900) further suggest that PCB/organochlorine pesticide (OCP) mixtures are positively associated with MASLD, with lifestyle factors modifying the risk. Collectively, these data support a link between environmentally relevant PCB exposures and hepatocellular injury and steatosis in the general population, with PCB-180 being identified as a possible sentinel marker for surveillance [25,26,27].

These associations are robust in models adjusting for cardiometabolic risk factors, and notably, the relationship is stronger for MASLD than for NAFLD under the older definition [25]. The recent literature data further highlight that PCBs, as endocrine-disrupting chemicals, can induce MASLD via nuclear receptor interference, mitochondrial dysfunction, and epigenetic modifications [28].

Epidemiological data from highly exposed human cohorts confirm a high prevalence of TASH, with strong associations between serum PCB levels, biomarkers of hepatocyte death (e.g., cytokeratin 18), insulin resistance, dyslipidemia, and proinflammatory cytokines [14,29]. Sex differences have also been observed, with some evidence suggesting females may be more sensitive to PCB-induced liver injury [30], although certain PCB congeners may exert greater hepatotoxicity in males, particularly in developmental exposures [26,31,32].

Populations at risk for developing TASH due to PCB exposure include individuals with high environmental or occupational exposure, such as residents living near former PCB manufacturing sites, workers in industries handling PCBs, and populations exposed through contaminated food or water sources [7].

Additional risk factors are present in individuals with metabolic comorbidities, specifically those with obesity, diabetes, or dyslipidemia, who demonstrate stronger associations between PCB exposure and liver injury biomarkers. Older adults may be at increased risk due to bioaccumulation over time, as shown in population-based studies [33,34]. The current lack of accessible and reliable biomarkers, however, could be counterbalanced by following more traditional ones in populations exposed to risk; Table 2 provides an overall summary of the most extensively studied indicators that may serve as potential clinical biomarkers for predicting the onset of PCB-associated liver disease.

### 4.1. Weight Loss in Patients with High Body Burdens of PCBs and Hepatic Injury

Several studies have demonstrated that, after bariatric surgery or intensive dietary interventions causing rapid or extensive weight loss, serum PCB concentrations increase significantly, up to 2–4% per kilogram of weight lost, and with sustained elevations for at least 12 months post-intervention [41,42].

From a pathophysiological point of view, during lipolysis, the stored lipophilic PCBs rapidly mobilize into the circulation, reaching the liver in very high concentrations, disrupting hepatic metabolism, inducing oxidative stress, and promoting steatohepatitis and hepatocellular injury [1,14].

Observational and mechanistic studies have shown positive correlations between increased serum PCB levels and liver toxicity markers, including transaminases and cytokeratin 18, independent of age and BMI. The risk is particularly relevant in individuals with high baseline PCB burdens, such as older adults or those with occupational or environmental exposures [43]. While the literature supports the plausibility of acute hepatic injury due to PCB mobilization during rapid weight loss, most studies are observational and neither establish direct causality nor quantify the risk of clinically significant acute liver injury. Nonetheless, the consistent association between increased circulating PCBs and hepatic dysfunction markers warrant caution and further investigation, especially in high-risk populations.

### 4.2. PCBs and “Lean MASLD”

“Lean MASLD”, i.e., the presence of steatosis in patients with BMI <25 and normal metabolic profiles, seems to be a distinct and challenging clinical phenotype. In this context, PCBs have been proposed as potential etiological factors specifically for those people who have no signs of metabolic disorders, particularly in the normal-weight population. In fact, PCBs bioaccumulate in the food chain, and exposed populations may come into contact with them through dietary ingestion or the incidental ingestion of soil or dust, with subsequent distribution primarily to the liver and adipose tissue [20].

Recent epidemiological and mechanistic studies have demonstrated that exposure to PCBs, particularly congeners such as PCB-180 and PCB-126, is associated with an increased risk of MASLD, independent of obesity or classic metabolic syndrome features [1], thus supporting the existence of a direct toxicant effect. PCBs acting as endocrine disruptors could, in fact, induce hepatic steatosis, inflammation, and fibrosis through the alteration of lipid metabolism, the activation of inflammatory pathways, and the modulation of nuclear receptors and microRNAs (miRNAs), even in the absence of metabolic syndrome. In particular, this theory has been validated in an animal model, with the observation that PCB exposure upregulated miR-155 and miR-34a, which induce the expression of proinflammatory cytokines and inflammation in the liver and reduce the expression of peroxisome proliferator-activated receptor α, which in turn, impairs lipid oxidation and hepatic steatosis [15].

## 5. Dioxin-Like PCBs (DL-PCBs) and Liver Diseases: Evidence from In Vitro and In Vivo Models

In recent years, in vitro cellular models have emerged as valuable tools for investigating the relationship between PCB exposure and the risk of developing or progressing to MASLD. These systems allow researchers to examine, under controlled conditions, key processes such as lipid accumulation, oxidative stress, and inflammatory responses, thereby helping to elucidate the molecular mechanisms by which PCBs may contribute to liver diseases. Similarly, numerous studies use in vivo animal models to validate and expand the results obtained in vitro in more realistic models, such as rats and mice. The hypothetical mechanisms by which PCBs determine hepatic steatosis, inflammation, fibrosis, and liver cancer are numerous and complex, as outlined in the next two sections.

### 5.1. DL-PCBs, Lipid, Glucose, Iron Metabolism, and Liver Carcinogenesis

DL-PCBs (such as PCB-126, PCB-169, PCB-81, PCB-77) exert their toxic effects primarily through the activation of AhR, a cytosolic ligand-activated transcription factor [44]. The canonical AhR response is the induction of cytochrome P450 (CYP) enzymes (e.g., CYP1A1, CYP1A2, CYP1B1). While this is a detoxification pathway, it also generates significant oxidative stress as a byproduct. More directly, these sustained enzyme activities can disrupt the normal metabolism of endogenous substrates, including lipids and steroids. Upon the binding of exogenous or endogenous ligands, AhR translocates to the nucleus and dimerizes with ARNT (aryl hydrocarbon receptor nuclear translocator), and this complex binds xenobiotic response elements (XREs) to induce target genes such as CYP1A1 [44].

In addition to xenobiotic metabolism, AhR signaling also intersects with immune regulation, barrier-tissue homeostasis, and redox pathways. Dysregulated or persistent activation (e.g., by DL-PCBs) can perturb metabolism, immunity, and normal tissue development.

#### 5.1.1. Lipid Metabolism Alterations

AhR activation can suppress the expression of genes involved in peroxisome proliferator-activated receptor α (PPARα) signaling. PPARα is the master regulator of fatty acid β-oxidation (the breakdown of fats in the mitochondria for energy production). When this pathway is suppressed, fatty acids (FAs) are shunted toward storage rather than oxidation. Additionally, AhR signaling can also suppress the synthesis and secretion of very-low-density lipoprotein (VLDL), the vehicle by which the liver exports triglycerides to peripheral tissues. When VLDL secretion is impaired, synthesized triglycerides become trapped within the hepatocytes, directly promoting steatosis.

A study using the human HepaRG liver cell model demonstrated that exposure to PCB-126, even at very low concentrations, disrupts lipid metabolism by reducing polyunsaturated fatty acids (PUFAs) and increasing sphingolipid levels. These changes were accompanied by oxidative stress, alterations in taurine metabolism, and the activation of AhR. The authors, using an integrated multi-omics approach, highlighted that docosatrienoate depletion was the most sensitive biomarker of PCB-126 exposure, and provided mechanistic insight into how this DL-PCB may promote NAFLD [45].

Furthermore, hepatocytes exposed to PCB-126 showed an increase in intracellular lipid accumulation compared to controls, as measured by Oil Red O extraction and colorimetry. In the corresponding rat animal model, a single dose of PCB-126 increased hepatic triglyceride content and led to the downregulation of the microsomal triglyceride transfer protein (MTP), along with the upregulation of sterol regulatory element-binding protein 1c (SREBP1c) and diacylglycerol O-acyltransferase 2 (DGAT-2). These in vivo results support and extend the in vitro observations, pointing to a dual mechanism of hepatic fat accumulation: impaired triglyceride secretion, reduced MTP expression, and enhanced triglyceride synthesis, via SREBP1c and DGAT-2 induction [46].

Emerging evidence indicates that DL-PCBs also exert profound epigenetic effects, altering miRNA expression and RNA methylation marks that regulate hepatic metabolism and fibrogenesis. Studies on animal models have shown that PCB-126 induces steatohepatitis and fibrosis by upregulating miR-155 and miR-34a, suppressing PPARα expression, and by impairing lipid oxidation and promoting hepatic lipid accumulation [15,47].

Recent work indicates that PCB-126 can reprogram post-transcriptional gene regulation in NASH models. It does so by altering transcriptome-wide RNA modification patterns and modulating genes involved in lipid metabolism, inflammation, and fibrosis, thus highlighting an additional epigenetic layer contributing to disease progression [48,49].

To confirm that AhR signaling mediates the metabolic toxicity of PCB-126, experiments have been conducted in AhR-deficient rats [50]. The characteristic disturbances in lipid homeostasis observed in wild-type animals were not observed in AhR-deficient rats. These findings solidify the central role of AhR in driving DL-PCB-induced hepatic metabolic reprogramming.

Combined exposure to the dioxin-like compounds TCDD, pentachlorodibenzofuran (PeCDF), and PCB-126 produces additive hepatotoxic effects marked by steatosis and inflammation. Short-term studies in mice showed AhR activation, lipid accumulation, and focal hepatic inflammation, particularly in females [51]. Long-term exposure in rats led to persistent steatosis, inflammatory lesions, bile-duct proliferation, and liver tumors [52]. Overall, these findings demonstrate that dioxin-like mixtures disrupt lipid metabolism and promote progressive liver injury, reinforcing the need for the cumulative risk assessment of AhR agonists.

In addition to hepatocellular effects, DL-PCBs such as PCB-77 also disrupt systemic lipid metabolism through extrahepatic mechanisms. In vitro, PCB-77 altered adipocyte differentiation and markedly increased the secretion of pro-inflammatory adipokines, while in vivo exposure promoted body-weight gain, adipocyte hypertrophy, dyslipidemia, and enhanced atherosclerosis in mice. These adipose changes parallel hepatic lipid accumulation and contribute to DL-PCB–driven metabolic dysfunction in MASLD [53].

Recent findings have expanded this evidence in relation to other dioxin-like congeners. In a high-fat-diet (HFD) mouse model, exposure to PCB-169 aggravated hepatic steatosis and increased the expression of lipogenic genes such as PPARγ and CD36, while downregulating oxidative metabolism markers, including PPARα and carnitine palmitoyltransferase 1 (CPT1). Transcriptomic analyses further indicated the activation of PPAR, xenobiotic, and retinol signaling pathways, linking PCB-169 exposure to profound lipid metabolism remodeling [54]. Altogether, these studies demonstrate that DL-PCBs, through AhR-dependent and secondary nuclear receptor interactions, disrupt lipid metabolism, establishing a mechanistic basis for their contribution to NAFLD development and progression.

#### 5.1.2. Glucose Metabolism (Gluconeogenesis/Insulin Resistance) Alterations

Mechanistic data for DL-PCBs demonstrate direct hepatic reprogramming of intermediary metabolism via AhR. PCB-126, the most potent DL-PCB, suppresses forskolin-induced phosphoenolpyruvate carboxykinase (PEPCK) expression and attenuates gluconeogenesis through AhR signaling, findings which have been replicated across cell systems and supported by antagonist and dose–response evidence [55]. Using primary mouse hepatocytes, Zhang and colleagues demonstrated that exposure to PCB-126 directly disrupts hepatic glucose metabolism. Treatment reduced the glycogen stores and suppressed lactate-driven gluconeogenesis without causing acute toxicity or altering lipid metabolism, through the induction of PEPCK, an effect reversed by an AhR antagonist and reproduced by other DL-PCBs [55]. These findings suggest that PCB-induced AhR activation may contribute to metabolic disturbances associated with NAFLD.

Moreover, PCB exposure results in the downregulation of protective transcription factors and interferes with insulin and leptin signaling, exacerbating metabolic dysfunction and liver injury, especially when combined with HFDs [56,57].

Vorrink and colleagues demonstrated that the in vitro PCB-126 exposure of HepG2 cells impaired the hypoxia response by reducing the hypoxia-inducible factor-1 α (HIF-1α) nuclear localization and attenuating hypoxia-regulated reporter activity, while functionally lowering the glucose consumption under hypoxic conditions. In vivo, male Sprague–Dawley rats exposed parenterally to PCB-126 also displayed reduced hepatocellular nuclear localization of HIF-1α, confirming that the hypoxia-signaling interference observed in vitro translates to the whole organism. Mechanistically, because HIF-1α requires ARNT to function, and ARNT is also an essential component of the AhR complex, PCB-126-mediated activation of AhR may compete with HIF-1α signaling, thereby compromising hepatocyte adaptation to hypoxia and potentially aggravating metabolic dysfunction [58].

Within the context of glucose metabolism, early disturbances induced by PCB-126 appear to precede the onset of hepatic steatosis. In male rats, PCB-126 exposure caused marked hypoglycemia through the suppression of gluconeogenesis and reduction in peroxisomal fatty acid β-oxidation, leading to an impaired hepatic energy balance before triglyceride accumulation occurred [59]. Complementary cellular experiments supported these findings, indicating that the disruption of glucose production is an initiating event in PCB-126-induced lipid dysregulation. Overall, these results identify PCB-126 as an early driver of metabolic dysfunction, linking altered glucose homeostasis to the development of NAFLD [59].

Extending these observations to other dioxin-like congeners, Eaton et al. reported that PCB-81 exhibits potent AhR binding and activation in human in vitro assays, in some cases exceeding the activity of PCB-126 [60]. Although primarily a receptor-binding study, the findings suggest that PCB-81 could exert similarly strong transcriptional effects on metabolic pathways governed by AhR, including those regulating glucose metabolism. Such evidence underscores the diversity in potency among DL-PCBs and reinforces the central role of AhR activation in hepatic metabolic dysfunction [60].

#### 5.1.3. Iron Homeostasis

An expanding body of research indicates that hepatic iron accumulation is a significant contributor to MASLD pathogenesis. Iron is an essential micronutrient involved in numerous biological processes, including oxygen transport, electron transfer, and enzymatic reactions. However, dysregulated iron homeostasis in the liver can lead to excessive free iron, which acts as a potent pro-oxidant. In the context of steatosis, where hepatocytes are already burdened with excess lipids, the generation of iron-mediated reactive oxygen species (ROS) can trigger lipid peroxidation, protein oxidation, and DNA damage, exacerbating hepatocellular injury and inflammation [61]. Steatotic hepatocytes exhibit impaired FA β-oxidation and electron transport chain activity, conditions that favor ROS production. Excess iron further destabilizes mitochondrial membranes and promotes the formation of lipid peroxides, creating a feed-forward loop that intensifies oxidative damage. Additionally, iron overload has been shown to impair the liver’s intrinsic antioxidant defenses, including reductions in glutathione levels and the activities of superoxide dismutase and catalase [62].

Both in vitro and in vivo findings have highlighted the pivotal role of lipocalin-2 (LCN2), an iron-binding protein, in PCB-induced liver injury. In HepG2 cells, PCB-126 exposure increased LCN2 expression along with lipid and iron accumulation, effects reversed by siRNA-mediated knockdown or treatment with recombinant fibroblast growth factor 21 (FGF21). Consistently, in an in vivo model, mice exposed to PCB-126 developed steatohepatitis and hepatic iron overload accompanied by elevated LCN2 levels, while FGF21 administration markedly improved these lesions. These results demonstrate that FGF21 mitigates PCB-induced NAFLD by modulating LCN2-dependent metabolic and inflammatory pathways, underscoring the central contribution of LCN2-driven inflammation to PCB-related liver damage [63].

Other in vitro experiments performed on HepG2 hepatocytes revealed that exposure to PCB-153 and PCB-126 markedly suppressed the expression of hepcidin, a central regulator of systemic iron metabolism. The repression was stronger with PCB-126 and was mechanistically linked to estrogenic activity, as both congeners acted through an estrogen response element (ERE) within the hepcidin promoter, mimicking the effect of 17β-estradiol. These results were confirmed in vivo, using wild-type mice. The administration of PCB-126 in mice caused the downregulation of hepatic hepcidin, accompanied by elevated serum iron and reduced iron content in the liver and spleen; notably, these changes did not occur in hepcidin-deficient mice. Additionally, hepatocytes in the livers of PCB-126-challenged mice accumulated severe lipid droplets, further linking iron dysregulation to steatotic changes. These findings demonstrate a novel mechanism by which PCBs can disrupt iron homeostasis and promote lipid accumulation at both the hepatocellular and organismal level [64].

Furthermore, PCB-126 is known to impair hepatic function; however, the precise mechanisms remain incompletely defined. Recent evidence suggests that ferroptosis, an iron-dependent form of regulated cell death characterized by uncontrolled lipid peroxidation, may contribute to PCB-126 induced hepatotoxicity [65,66]. In this context, PCB-126 mediated the suppression of hepcidin, and a consequent increase in hepatic iron availability sensitized hepatocytes to ferroptotic injury.

#### 5.1.4. Liver Carcinogenesis

Beyond metabolic dysregulation, DL-PCBs have also been implicated in tumor-related processes of hepatocellular carcinoma (HCC). In HCC cell models, PCB-126 was shown to promote epithelial–mesenchymal transition (EMT), a key step in tumor progression characterized by reduced E-cadherin and increased N-cadherin and vimentin. Mechanistically, PCB-126 activated the pyruvate kinase M2 (PKM2)/signal transducer and activator of transcription 3 (STAT3)/Snail1 signaling axis through AhR- and estrogen receptor-dependent pathways [67]. These findings indicate that PCB-126 can enhance pro-tumorigenic processes in HCC, linking DL-PCB exposure not only to steatosis but also to pathways involved in liver cancer progression.

In another study using a Kras-transgenic zebrafish model, PCB-126 was identified as a pollutant capable of inducing hepatic inflammation and promoting early liver tumorigenesis. PCB-126 increased neutrophil infiltration and oncogenic liver growth, demonstrating pro-tumorigenic activity [68]. This work further highlights PCB-126’s contribution to the initiation of inflammation-driven liver cancer.

### 5.2. DL-PCBs, Oxidative Stress, and Inflammation

DL-PCBs profoundly alter hepatic and systemic energy metabolism through AhR-dependent mechanisms. Such metabolic disruptions not only initiate lipid accumulation and glucose imbalance but also set the stage for secondary processes, including oxidative stress and inflammation, which further drive MASLD progression.

A metabolomics-driven mouse study showed that PCB-126 elicited robust oxidative stress and redox imbalance, effects that were markedly amplified when pre-existing liver injury was present. Across control and NASH-like models (methionine-choline deficient diet-MCD), PCB-126 consistently perturbed glutathione/thiol metabolism and mitochondrial-linked lipid oxidation products (e.g., 4-idrossi-2-nonenale-glutatione (4-HNE-GSH), oxylipids), while downregulating hepatic redox-responsive genes. These findings position oxidative stress as a core feature of DL-PCB toxicity and suggest heightened vulnerability in diseased livers [3].

Furthermore, in vitro evidence in HepG2 cells revealed that PCB-126 activates mitogen-activated protein kinase (MAPK) pathways, including extracellular signal-regulated kinases 1 and 2 (ERK1/2), c-Jun N-terminal kinase (JNK), and p38, in a dose- and time-dependent manner. This activation was associated with increased oxidative stress and suggested a link between ROS generation and MAPK-driven inflammatory signaling. Together, these findings established oxidative stress and MAPK pathway activation as interconnected mechanisms underlying PCB-126-induced hepatic injury [69].

Similarly, PCB-169 exposure aggravated HFD-induced hepatic injury in male C57BL/6 mice, intensifying inflammatory responses. The treatment increased the hepatic expression of pro-inflammatory cytokines (tumor necrosis factor-α (TNF-α), interleukin-1β (IL-1β), interleukin-6 (IL-6)) and oxidative stress markers while activating AhR and PPAR-related pathways. These results highlight the potent pro-inflammatory nature of DL-PCBs and their contribution to liver injury progression [54].

Among DL-PCBs, PCB-77 has been shown to exert marked oxidative-stress-mediated toxicity. In HepG2 cells, PCB-77 induces apoptosis through the activation of the AhR/ARNT–CYP1A1 axis, leading to ROS generation and a distinct pro-apoptotic transcriptional signature. Consistently, in vivo studies in rats demonstrated that PCB-77 rapidly induced hepatic cytochrome P450 enzyme activation and promoted lipid peroxidation, confirming its ability to trigger early oxidative damage in the liver. Overall, these findings indicate that PCB-77 elicits hepatotoxicity through AhR-dependent pathways and oxidative-stress mechanisms characteristic of DL-PCBs [70,71].

**Figure 1:**
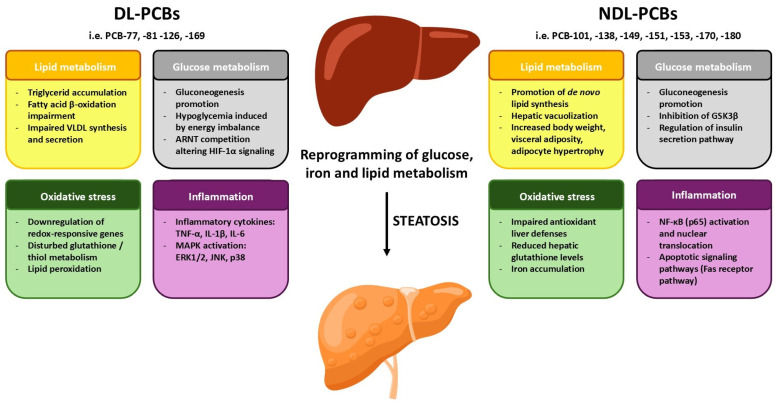
Schematic representation of all molecular mechanisms modulated by DL-PCBs and NDL-PCBs. This schematic represents a generic liver cell and provides a conceptual overview of the main molecular and cellular pathways involved. Abbreviations: ARNT, Aryl Hydrocarbon Receptor Nuclear Translocator; ERK1/2, Extracellular signal-Regulated Kinase 1 and 2; GSK3β, Glycogen Synthase Kinase 3 β; HIF-1α,Hypoxia-Inducible Factor-1 α; IL-1β, Interleukin-1β; IL-6, Interleukin-6; JNK, c-Jun N-terminal kinase; MAPK, Mitogen-Activated Protein Kinase; NF-κB), Nuclear Factor Kappa B; TNF-α, Tumor Necrosis Factor α;VLDL, Very-Low Density Protein.

The molecular mechanisms modulated by DL-PCBs are summarized in Figure 1 and the involved mechanistic pathways in Table 3.

## 6. Non-Dioxin-Like PCBs (NDL-PCBs) and Liver Steatosis: In Vitro and In Vivo Studies

### 6.1. NDL-PCBs and Xenobiotic Receptor Modulation in Lipid and Glucose Metabolism

NDL-PCBs constitute the majority of the 209 PCB congeners, including PCB-28, PCB-52, PCB-101, PCB-138, PCB-153, and PCB-180. Studies have shown that PCB-153 and PCB-180 are particularly widespread in the environment and display a pronounced tendency to bioaccumulate in human serum and adipose tissue, often reaching higher concentrations than many dioxin-like congeners [European Food Safety Authority (EFSA), 2010; Agency for Toxic Substances and Disease Registry 2022 (ATSDR)]. A substantial body of evidence indicates that NDL-PCBs contribute to the development of hepatic steatosis through multiple mechanistic pathways, including the impairment of mitochondrial bioenergetics, the induction of oxidative stress, and the dysregulation of lipid and energy homeostasis [72] (ATSDR, 2022). Unlike DL-PCBs, NDL-PCBs exhibit minimal AhR activation and instead act as potent activators of nuclear receptors (NRs), a transcription factor superfamily which regulates lipid metabolism, metabolic homeostasis, and pathways involved in protecting against xenobiotic stressors [7,73]. In recent years, increasing attention has been directed toward a subgroup of NRs, so-called xenobiotic receptors (XRs), which are enriched in the liver and intestine.

XRs orchestrate cellular responses to harmful compounds, including environmental pollutants and their metabolites, by activating the expression of genes involved in phase I and phase II drug-metabolizing enzyme detoxification pathways and transporter systems [74]. It has been demonstrated that NDL-PCBs interact with the two prominent members of the XR family: the pregnane X receptor (PXR, also called SXR) and the constitutive androstane receptor (CAR).

PXR plays a crucial role in regulating lipid metabolism, fatty acid uptake, triglyceride storage, and cholesterol homeostasis [75]. Studies in mouse cells have shown that ligand binding activates PXR, promoting its translocation into the nucleus, where it induces the expression of genes encoding enzymes involved in all three phases of xenobiotic metabolism: phase I enzymes, such as cytochrome P450 (CYP), phase II conjugating enzymes, such as glutathione S-transferase (GST), and phase III drug transporters, through direct binding to pregnane X receptor response elements (PXREs) and through interactions with other nuclear receptors, such as retinoic acid receptors (RARs) and retinoid X receptors (RXRs) [76].

On the other hand, CAR influences gluconeogenesis, lipogenesis, and bile acid metabolism. CAR is essential for liver detoxification by inducing enzymes and transporters involved in processing drugs, bilirubin, and bile acids. In the inactive form, it is associated with the chaperone heat shock protein 90 (Hsp90) and cytoplasmic CAR retention protein (CCRP) in the cytoplasm. Upon ligand binding, protein phosphatase 2A (PP2A) is recruited and dephosphorylates CAR, promoting its dissociation from the chaperone complex. The activated receptor then moves into the nucleus, where it heterodimerizes with RXR and binds to the phenobarbital-responsive enhancer module (PBREM) to regulate the transcription of target genes, including phase I and phase II drug-metabolizing enzymes as well as factors governing cell proliferation and apoptosis, thus playing a central role in xenobiotic responses [44].

Although PXR and CAR generally contribute to hepatic metabolic homeostasis, there is growing evidence indicating that their dysregulation can promote hepatic steatosis pathology. Indeed, the constitutive activation of PXR in mice induces marked hepatic steatosis driven by excessive triglyceride deposition, whereas PXR-deficient mice display significantly reduced hepatic lipid accumulation, underscoring its central role in triglyceride homeostasis and steatosis development [77,78]. Indeed, PXR can regulate CD36 directly or through the activation of PPARγ to increase the flow of FAs into hepatocytes [79]. Moreover, the induction of PXR expression drives the overexpression of CYP3A11I, a gene associated with NAFLD progression [80]. Similarly, the aberrant activation of CAR has been implicated in lipid metabolic disturbances [81].

Recent studies have shown that interactions with NDL-PCBs impair the liver’s protective responses, promoting de novo lipid synthesis and triggering adverse outcome pathways (AOPs) associated with the development of hepatic steatosis [35,82].

Salman and Plant demonstrated that the non-coplanar PCBs-138, -153, -180, and -194 can bind to both XRs [83]. In terms of CAR modulation, in vitro studies performed in Alpha mouse liver 12 (AML-12) cells and human HepG2 cells have shown that NDL congeners, such as PCB-3, PCB-6, PCB-8, PCB-9, PCB-138, PCB-149, PCB-151, PCB-153, PCB-170, PC-174, PCB-180, and PCB-187, indirectly activate mouse and human CAR by binding to the epidermal growth factor receptor (EGFR). Among these, PCB-151, -153, -170 and -180 have shown a higher affinity for the receptor binding. The NDL-PCBs–EGFR interaction prevents binding of the EGF ligand to its receptor. Consequently, NDL-PCBs disrupt the EGFR signaling cascade. Specifically, PCB-induced activation of EGFR reduces phosphorylation of CAR, resulting in CAR activation. This event is accompanied by decreased phosphorylation—and thus inactivation—of AKT, ultimately impairing the PI3K/AKT/mTOR signaling pathway. Dephosphorylation and inactivation of AKT and mTOR are associated with type II diabetes, related metabolic disorders, and hepatic insulin resistance. Indeed, growing evidence indicates that NDL-PCB-mediated disruption of the AKT/mTOR pathway impairs insulin-stimulated AKT phosphorylation and downstream mTOR activity. As a result, NDL-PCBs compromise insulin signal transduction, promoting increased gluconeogenesis, dysregulated lipid metabolism, and elevated hepatic glucose production. Because insulin resistance is a hallmark of MASLD, NDL-PCB-induced AKT/mTOR dysfunction represents a key mechanistic link between environmental pollutant exposure and MASLD pathogenesis [35,84,85].

Similar data were observed in the C57Bl/6J in vivo model. Animals co-exposed to an HFD (high fat diet) and gavage of 20 mg/kg of Aroclor 1260 (an NDL-PCB congener mixture that closely reflects the PCB mixtures typically found in the environment and in human tissues) have shown the induction of the liver CYP2B10 gene consistent with CAR activation and the development of steatohepatitis. Aroclor 1260 also reduced the phosphorylation of hepatic AKT and mTOR in a diet-independent manner in vivo; instead, in mice given an HFD, the expression of hepatic STAT3, a critical anti-inflammatory signal in NAFLD and liver fibrosis, decreased. In addition, Aroclor 1260 induced high levels of circulating ALT, AST, and FGF21 and increased vacuolization/lipid accumulation in an HFD mouse in vivo model [29,84,86,87].

Due to its persistence in the environment and bioaccumulation in human tissues, PCB-153 has been extensively studied. This congener has been demonstrated to be the most potent activator of PXR and CAR receptors and to induce the over-expression of CYP3A enzymes. This modulation can alter the metabolism of endogenous substrates and co-exposed chemicals, potentially influencing toxicity outcomes. Through sustained activation of these pathways, PCB-153 contributes to long-term disturbances in hepatic detoxification systems and overall metabolic homeostasis [79,88].

Peiwen and colleagues demonstrated that PCB-153 induces glucose metabolic dysregulations in HepG2 cells, enhancing the expression of glucose-6-phosphatase catalytic subunit 1 (G6PC) and PEPCK enzymes in promoting gluconeogenesis and inhibiting glycogen synthase kinase 3β (GSK3β), which plays a key role in suppressing glycogen synthesis [89]. In addition, DNA methylome analysis indicated significant alterations across diverse biological pathways, including morphine addiction, insulin secretion, oxytocin signaling, inflammatory mediator regulation of transient receptor potential (TRP) channels, cyclic adenosine monophosphate (cAMP) signaling, adipocyte lipolysis regulation, cell-cycle control, efferocytosis, cell adhesion molecules, and autophagy [89,90].

A substantial body of evidence also indicates that PCB-153 functions as a diet-dependent obesogen, exacerbating NAFLD-related dysfunctions. Studies in male C57BL/6J mice co-exposed to an HFD and gavage with PCB-153 showed increased body weight, visceral adiposity, adipocyte hypertrophy, hepatic lipid accumulation, and elevated serum AST and ALT levels. Likewise, co-exposure impaired the expression of genes closely related to fatty acid β-oxidation, such as PPARα, CPT1A, and CPT2, while increasing the expression of fatty acid synthase (FAS), a key regulator of lipid biosynthesis [44,91].

Liu and colleagues investigated the effects of intraperitoneal PCB-153 exposure in a Sprague–Dawley rat model. They reported that PCB-153 increased liver weight, induced oxidative stress through the suppression of glutathione peroxidase (GPx1) and superoxide dismutase (SOD), and promoted hepatotoxicity characterized by impaired apoptosis via Nuclear Factor kappa B (NF-κB)) activation and caspase inhibition. Additional involvement of the PI3K/AKT and ERK signaling pathways was also observed [92].

The effects of NDL-PCBs on lipid metabolism were also examined in adipocytes by Del Piano and co-workers. Specifically, mature 3T3-L1 mouse adipocytes were exposed to PCB-101, -153, or -180, and the expression of the aquaglyceroporins AQP3, AQP7, and AQP9, key mediators of glycerol release and uptake in adipose tissue, respectively, was assessed. Data highlighted the ability of NDL-PCBs to modulate aquaglyceroporin expression. Furthermore, PCB-153 increased adipocyte lipid accumulation, altering the expression of enzymes involved in glycerol metabolism and lipid accumulation such as PPARγ, fatty acid binding protein 4 (Fabp4), glycerol kinase (Gyk), diacylglycerol acyltransferase 1 (Dgat1), and acylglycerol-3-phosphate O-acyltransferase 9 (Agpat9) [93].

Finally, a recent study integrating in vitro HepG2 cell models with machine learning and epidemiological data revealed that acute exposure to PCB-153 disrupted glucose metabolism through mitochondrial dysfunction. Specifically, PCB-153 impaired mitochondrial function, lowering the mitochondrial DNA (mtDNA) copy number, adenosine triphosphate (ATP) production, membrane potential, and ATPase activity, while increasing ROS and altering mitochondrial morphology [89].

### 6.2. The Role of NDL-PCBs in Oxidative Stress, Inflammation, and Iron Homeostasis

In the liver, ROS are generated not only through normal metabolic processes but also during the detoxification of xenobiotics. When the redox equilibrium is disturbed, oxidative stress develops, impairing hepatic function, influencing inflammatory signaling, and driving the etiopathogenesis of liver diseases such as MASLD.

NDL-PCB congeners such as PCB-153 and Aroclor 1260 have been shown to impair antioxidant liver defenses. In this context, different research projects have aimed to clarify the role of NDL-PCB exposure in oxidative stress and inflammation processes. Treatment of metabolically competent human HepG2 liver cells with high concentrations of PCB-153 and the co-planar PCB-77 showed significant apoptotic changes. In particular, microarray and in silico analyses highlighted that PCB-77 has a strong toxic effect on hepatocytes, whereas PCB-153 modulates TNF-α receptor signaling, leading to oxidative stress through the involvement of metallothionein gene families and inducing apoptosis by the Fas receptor signaling pathway [7]. In vitro and in vivo (AML-12 hepatocytes, 3T3-L1 adipocytes, and C57 male mice, respectively) studies performed by Wu and co-workers also demonstrated that PCB-153 induced the activation and nuclear translocation of p65 NF-κB) and the expression of inflammatory markers IL-1β and IL-6. PCB-153 also decreased the expression of hepatocyte nuclear factor 1β (HNF1β) and GPx1, two crucial redox regulators involved in maintaining ROS balance [94]. NDL-PCBs also interfere with the functional activity of hepatic transcription factors, nuclear factor erythroid 2-related factor 2(NRF2). NRF2 is a key regulator of cellular defenses, driving the expression of anti-inflammatory, antioxidant, and cytoprotective genes. Numerous studies have shown that altering its activity—whether promoting or suppressing it—strongly influences the progression of liver diseases [56]. Proteomic analyses have shown that exposure to Aroclor 1260 downregulates NRF2, leading to reduced hepatic glutathione levels and increasing the liver’s susceptibility to oxidative stress [57]. A recent study performed on steroid and xenobiotic receptor knockout (SXRKO) mice, with PXR knock-out, which were chronically or perinatally exposed to a low dose of PCB-153 revealed elevated oxidative stress, symptoms of hemolytic anemia, and premature death in perinatally exposed animals. Instead, chronic exposure induced the accumulation of the 3-OH-PCB-153 metabolite, lower levels of blood hemoglobin (HGB), and the occurrence of intestinal tumors in adult animals compared to wild-type animals [95].

In a recent work, Kim and co-workers have shown that intraperitoneal exposure to DL-PCB-126 or NDL-PCB Aroclor 1260 induces liver damage, hepatic steatosis, inflammation, and hepatic iron overload (HIO) in an HFD male C57Bl/6 mouse model. The authors also demonstrated that Aroclor 1260 reduces the expression of hepatic six transmembrane protein of prostate 2 (STAMP2), a metalloreductase which is highly expressed in the liver and which plays a central role in iron and copper reduction, redox regulation, and metabolic homeostasis, and which was significant reduced in NAFLD in vivo and in vitro models [29]. Although oxidative stress and inflammatory signaling represent central mechanisms of PCB-induced liver injury, the estrogen-related effect of these compounds is another means of impacting liver homeostasis. While endogenous estrogens are generally considered hepatoprotective, limiting lipid accumulation and oxidative stress, several PCBs show estrogenic or anti-estrogenic activity through partial binding to estrogen receptors [96,97]. Importantly, PCBs do not act as classical estrogens nor as Selective Estrogen Receptor Modulators (SERMs), but rather as endocrine disruptors. In the liver, the predominant toxic mechanisms of both DL-PCBs and NDL-PCBs are driven by oxidative stress and inflammatory signaling, particularly involving AhR activation, which can counteract estrogen receptor pathways [98]. Within this altered redox and signaling environment, estrogen receptor-dependent transcriptional targets that are closely linked to iron metabolism and oxidative balance, such as hepcidin, become particularly susceptible to dysregulation following PCB exposure.

It is interesting to note that PCBs also interfere with the hepcidin–ferroportin (FPN) axis. Hepcidin is a master regulator of dietary iron absorption and iron egress from macrophages. Dysregulation in hepcidin expression gives rise to disordered iron homeostasis. Qian and colleagues observed that in vitro exposure of HepG2 cells either to NDL-PCB-153 or DL-PCB-126 greatly impaired hepcidin expression. Indeed, both PCB-153 and PCB-126 exhibited estrogen-like activity, with PCB-126 showing a more potent effect than PCB-153 in HepG2 cells. Mechanistic analyses further revealed that these PCBs repressed hepcidin transcription by acting on a functional ERE within the hepcidin promoter, mirroring the regulatory effects of 17β-estradiol. Consistent with these in vitro findings, hepatic hepcidin expression was also reduced in wild-type mice following PCB-126 exposure [64].

### 6.3. NDL-PCBs and Thyroid Hormone Disruption in Hepatic Steatosis

Thyroid hormones (THs), primarily thyroxine (T4) and triiodothyronine (T3), play a critical role in regulating systemic metabolism, including hepatic lipid homeostasis. They modulate key processes such as FA β-oxidation, lipogenesis, and cholesterol metabolism in hepatocytes. Dysregulation of thyroid function, including hypothyroidism or low circulating T3 levels, has been increasingly associated with the development of NAFLD and hepatic steatosis. Mechanistically, impaired TH signaling reduces mitochondrial FA β-oxidation and promotes lipid accumulation in hepatocytes, thereby contributing to liver injury and inflammation [99,100].

Clinical and epidemiological studies have consistently shown that patients with subclinical or overt hypothyroidism have a higher prevalence of hepatic steatosis, highlighting the importance of thyroid–liver crosstalk in metabolic liver disease [101].

NDL-PCBs may contribute to the development and progression of MASLD by disrupting TH homeostasis and signaling in the liver. Indeed, thyroid hormones are central regulators of hepatic lipid metabolism, promoting mitochondrial FA β-oxidation via PPARα activation, suppressing de novo lipogenesis through the inhibition of SREBP-1c, and facilitating triglyceride export as very-low-density lipoproteins [102].

Growing evidence indicates that the thyroid-disrupting effects of NDL-PCBs are primarily mediated by their hydroxylated and sulfated metabolites, which act through complementary and reinforcing mechanisms. Hydroxylated PCBs display strong structural similarity to thyroid hormones and bind transthyretin (TTR) with affinities that often exceed those of T4, thereby displacing the hormone and altering its systemic and tissue-specific distribution. Sulfated metabolites, once considered mere detoxification products, are now recognized as biologically active intermediates that prolong and amplify thyroid disruption. Together, these metabolites impair thyroid hormone homeostasis by interfering with deiodinase activity and sulfation pathways, ultimately reducing T4 and T3 bioavailability [103].

Concurrently, hydroxylated and sulfated NDL-PCB metabolites strongly induce hepatic phase I and phase II detoxification enzymes, thereby accelerating TH metabolism and clearance [104,105,106]. At the cellular level, these metabolites potently suppress thyroid hormone receptor (TR)-dependent transcription in vitro, disrupting T3-regulated expression of genes involved in fatty acid β-oxidation, mitochondrial function, and lipid export, with the parent NDL-PCBs contributing to a lesser extent [107,108].

In vivo studies in rat and mouse models have demonstrated that exposure to PCB-153, Aroclor 1254, and Aroclor 1242 reduced circulating levels of T4 [107]. Furthermore, PCB-180 has been shown to activate hepatic metabolic enzymes, including UDP-glucuronosyltransferases (UGTs) and cytochrome P450 (CYP) enzymes, thereby increasing the oxidative catabolism of thyroid hormones in the liver [109].

All the molecular mechanisms modulated by DL-PCBs and NDL-PCBs are summarized in Table 4 and Figure 1.

## 7. PCBs-Induced Gut Dysbiosis and “Leaky Gut”

Recent evidence demonstrates that PCBs can induce gut dysbiosis and increase intestinal permeability (“leaky gut”) through several mechanisms. In animal models, oral exposure to environmental PCB mixtures alters the abundance and function of specific gut microbial taxa, with both parent PCBs and their metabolites directly influencing the composition of the gastrointestinal microbiota [110].

Human data remain limited; however, cross-sectional studies in exposed populations (e.g., children in e-waste areas) show that PCBs exposure is associated with significant alterations in gut microbiota composition. These changes, together with compromised intestinal barrier integrity, may contribute to systemic inflammation and increased metabolic disease risk, with important implications for human health [111]. A growing body of experimental evidence indicates that DL-PCBs can perturb the gut and intestinal barrier homeostasis, thereby providing a mechanistic link between environmental exposure and hepatic inflammation through the gut–liver axis [35,112]. In murine models, oral exposure to PCB-126 significantly alters gut microbial composition and diversity while increasing intestinal and systemic inflammatory markers, suggesting that DL-PCB toxicity may be initiated at the level of the gastrointestinal tract [113]. Consistent with this concept, manipulation or depletion of the gut microbiota markedly modifies PCB-induced metabolic fingerprints and hepatic transcriptomic responses, demonstrating that the microbiome critically shapes downstream hepatic toxicity [114]. Exposure to another DL-PCB congener, PCB-77, similarly induces reproducible shifts in gut microbial communities and is associated with the development of obesity, hyperlipidemia, hepatic lipid accumulation, and liver injury in mice, further supporting a conserved capacity of DL-PCBs to disrupt host–microbiome interactions and promote metabolic and hepatic dysfunction [115]. Importantly, early-life exposure to PCB-126 induces persistent activation of AhR and long-lasting remodeling of the gut microbiome, with greater alterations in microbial community structure, metabolic capacity, and functional pathways detectable in adulthood. These microbiome changes are accompanied by durable disturbances in hepatic amino acid and increased hepatic lipogenesis, indicating that transient developmental exposure can program long-term metabolic susceptibility via microbiome-mediated mechanisms [116]. PCB-126–induced dysbiosis has been also associated with increased abundance of Gram-negative bacteria, elevated luminal and systemic levels of lipopolysaccharide (LPS), and activation of Toll-like receptor 4 (TLR4)-dependent signaling pathways, together with evidence of impaired intestinal barrier integrity [117]. These alterations are consistent with the development of increased intestinal permeability, facilitating the translocation of bacterial endotoxins, such LPS, into the portal circulation, which has been implicated in chronic liver inflammation [118]. Even modest increases in portal LPS can activate innate immune responses in the liver via TLR4 on Kupffer cells and other non-parenchymal hepatic cells, thereby amplifying inflammatory and fibrogenic pathways [119]. Although direct data for PCB-169 and PCB-81 in the context of microbiome remodeling and endotoxemia remain limited, their shared ability to activate the AhR, a key regulator of intestinal epithelial integrity and mucosal immune homeostasis, supports a biologically plausible role for these congeners in promoting gut barrier dysfunction and endotoxin-driven hepatic inflammation [120].

The effects of NDL-PCBs on the gut microbiota have also been investigated. It has been demonstrated that oral exposure to PCB-153 impaired gut microbiota reducing the relative abundance of Bacteroidetes while increasing Firmicutes, along with a marked enrichment of *Proteobacteria*, *Actinobacteria*, *Saccharibacteria*, and *Deferribacteres*.These microbiota changes were associated with adverse host outcomes, including increased obesity, hepatic lipid accumulation, abdominal fat deposition, and dyslipidemia, suggesting a strong link between PCB-153–induced microbiome dysregulation and metabolic risk factors [121]. Gestational exposure of BALB/c mice to PCB-52 administered by oral gavage has been shown to induce not only maternal liver damage but also disruption of the intestinal barrier and alterations in the fecal gut microbiota of offspring [122]. Consistently, a recent study in male and female Sprague–Dawley rats reported that sub-acute inhalation exposure to PCB-52 did not affect overall microbial diversity but induced sex-specific and region-dependent shifts in gut microbiota composition. Notably, PCB-52 inhalation altered *Malassezia restricta* in the female jejunum, indicating that inhaled PCBs affect the broader gut microbial ecosystem beyond bacteria. Moreover, PCB-52 exposure disrupted predicted microbial functions, including chemotaxis, quorum sensing, protein synthesis, and energy metabolism, and revealed sex- and region-specific associations between PCB-52 metabolites and distinct microbial taxa along the gastrointestinal tract [110].

A recent study has also investigated the effect of PCB mixtures on the gut microbiome. Interestingly, Cheng and colleagues evaluated the effects of different concentrations of PCB mixtures (Aroclor 1242, 1248, 1254, and 1260) on the gut microbiome in mice. Authors observed that low-dose exposure increased bile acid-metabolizing bacteria (*Akkermansia muciniphila*, *Clostridium scindens*, and *Enterococcus*) in the large intestine and elevated bile acid levels in serum and the small intestine. In contrast, high-dose exposure did not alter bile acid levels but induced hepatic efflux transporters and increased ileal fibroblast growth factor 15 (FGF15), a key regulator of bile acid synthesis, cholesterol homeostasis and glucose metabolism. Overall, these findings indicated that PCB mixtures disrupt bile acid homeostasis in a dose-dependent manner through interactions between the gut microbiota and the gut–liver axis [123].

## 8. The Additive Effects of Different PCBs in Liver Diseases

A mixture of DL- and NDL-PCBs represent a real-life scenario that can impair hepatic function through complementary mechanisms: DL-PCBs primarily promote lipogenesis through AhR activation, while NDL-PCBs disrupt mitochondrial activity and energy homeostasis. When combined, these pathways may act synergistically, producing a more severe steatotic phenotype than either class alone. Furthermore, oxidative stress generated by NDL-PCB-induced mitochondrial ROS can deplete cellular antioxidant defenses, increasing susceptibility to the pro-oxidant effects of DL-PCBs and thereby amplifying liver injury [72,124] (EFSA, 2005; ATSDR, 2022).

Additional additive effects thathave been described include alcohol exposure and DL-PCB exposure: PCB-126 exacerbates alcohol-associated liver injury, amplifying steatosis, hepatomegaly, and transcriptional signatures consistent with impaired lipid oxidation and export. These “multiple hit” interactions likely operate through convergent mitochondrial dysfunction, redox stress, and impaired AMPK-CREB signaling, processes broadly implicated in progression from steatosis to steatohepatitis and fibrosis [125].

Beyond alcohol, dietary excess constitutes another important “multiple hit” that interacts with PCB mixtures. In HFD models, exposure to NDL-PCB-153 and PCB mixtures such as Aroclor 1260 markedly exacerbated hepatic steatosis, altered adipokine signaling, and worsened steatohepatitis severity, with DL congeners like PCB-126 further modifying the hepatic proteome and amplifying inflammatory and fibrotic pathways [54,91,126]. Recent data also show that PCB-169 aggravated diet-induced NAFLD and activated PPAR, arachidonic acid, and xenobiotic-metabolizing pathways, supporting the concept that multiple DL congeners can potentiate diet-driven MASLD progression.

At the organelle level, PCB exposures (both DL and NDL) are linked to mitochondrial injury, increased ROS production, and altered lipid droplet dynamics—lesions strongly tied to disease progression in MASLD. While mitochondria-centric reviews synthesize multi-toxicant evidence, PCB-specific models align with these signatures: decreased oxidative phosphorylation capacity, altered FA β-oxidation, and activated cell-death programs (including emerging ferroptosis links with PCB-126). These mechanisms explain how low-dose chronic exposures can shift hepatic set-points towards lipid retention, inflammation, and fibrogenesis, particularly in those with underlying metabolic risks [65,127,128].

## 9. Conclusions and Future Directions

The literature data reported in this review highlights the relevance of PCBs in the induction and worsening of liver diseases.

From a risk-assessment and policy perspective, several considerations are particularly relevant for clinicians and researchers. First, reliance on TEQ alone likely underestimates human PCB-related risk, because TEFs apply only to DL congeners, whereas epidemiological data consistently link both DL- and NDL-PCBs to altered liver enzymes and adverse hepatic outcomes. Second, effect sizes are clearly modified by co-exposures and susceptibility factors—such as diet quality, alcohol consumption, and diabetes/obesity—underscoring the need for mixture-aware analytical approaches and stratified analyses in human studies. Third, the receptor pharmacology of NDL-PCBs (including PXR/CAR activation and PPARα antagonism by PCB mixtures) suggests potential drug–toxicant interactions and variability in therapeutic responses (e.g., to PPARα agonists) among exposed individuals.

Key knowledge gaps include the lack of prospective repeated-measure cohorts with rigorously adjudicated MASLD/MASH endpoints; the need for more refined exposure assessment that distinguishes DL from NDL burdens and accounts for relevant metabolites; and the scarcity of human translational studies integrating receptor activation readouts (AhR/PXR/CAR) with lipidomics and mitochondrial function. As MASLD prevalence continues to rise, incorporating environmental exposure assessment into routine hepatology—particularly for patients with unexplained steatosis, persistent ALT elevation, or suboptimal response to therapy—may sharpen both preventive strategies and personalized treatments.

## Figures and Tables

**Table 1:** Classification, primary targets/pathways, and biomarkers for PCBs.

Congener(s)	Class	Primary Molecular Targets/Pathways	Typical Biomarkers (Examples)	Notes
PCB-77, -81, -126, -169 (non-ortho)	DL	AhR → DRE transcription; oxidative stress; lipid/glucose reprogramming	↑ CYP1A1, CYP1B1; AHRR; NQO1; UGT1A family	Assigned WHO TEFs; PCB-126 most potent among PCBs
PCB-105, -114, -118, -123, -156, -157, -167, -189 (mono-ortho)	DL	AhR (lower potency); partial DRE activation	Modest ↑ CYP1A1/1B1; AHRR	Have TEFs but far less potent than non-ortho DL congeners
PCB-28, -52, -101, -138, -153, -180 (ICES-6/indicator set)	NDL	PXR/CAR activation; endocrine and metabolic modulation	↑ CYP3A (e.g., CYP3A4), ↑ CYP2B (e.g., CYP2B6); ↑ ABCB1	No TEFs; dominate human body burden; key in surveillance

The arrows indicate: → induction; ↑ up-regulation.

**Table 2:** Summary of the potential clinical biomarkers for predicting the onset of PCB-associated liver disease.

Category	Biomarker	Biological Matrix	Biological Relevance/Endpoint	References
Exposure biomarkers	Total PCBs (lipid-adjusted)	Serum/plasma	Internal dose and body burden of PCBs	[7]
	PCB-126, PCB-118 (DL)	Serum/plasma	AhR activation, hepatotoxicity	[35]
	PCB-138, PCB-153, PCB-180 (NDL)	Serum/plasma	Metabolic and lipid dysregulation	[7]
Liver injury markers	ALT, AST	Serum	Hepatocellular injury (non-specific)	[7]
	GGT	Serum	Sensitive marker of environmental toxicant-related liver dysfunction	[7]
Hepatocyte cell death	CK18 M30	Serum	Hepatocyte apoptosis	[36]
	CK18 M65	Serum	Total hepatocyte cell death (apoptosis + necrosis)	[36]
Circulating microRNAs (liver-specific)	miR-122-5p	Serum/plasma	Hepatocellular injury and steatosis	[29]
Circulating microRNAs (stress/apoptosis)	miR-34a-5p	Serum/plasma	Apoptosis, oxidative stress, and NAFLD/TASH-like injury	[15]
Circulating microRNAs (inflammation)	miR-155-5p	Serum/plasma	Macrophage activation and inflammatory signaling	[15]
	miR-21-5p	Serum/plasma	Fibrogenesis and tissue remodeling	[29]
	miR-146a-5p	Serum/plasma	Regulation of inflammatory response (AhR-related pathways)	[29]
Inflammatory markers	TNF-α	Serum	Kupffer cell activation and chronic inflammation	[15]
	IL-6	Serum	Systemic and hepatic inflammation	[15]
	MCP-1 (CCL2)	Serum	Monocyte recruitment and liver inflammation	[37]
Oxidative stress markers	GSH/GSSG ratio	Serum/liver tissue	Redox imbalance induced by PCB exposure	[3]
	Malondialdehyde (MDA)	Serum	Lipid peroxidation	[3]
Metabolic dysfunction	Triglycerides	Serum/liver tissue	Hepatic steatosis	[38]
	Adiponectin	Serum	Metabolic dysregulation and insulin resistance	[39]
	HOMA-IR	Serum	Insulin resistance associated with PCB exposure	[40]

**Table 3:** Key mechanistic pathways involved in DL-PCBs-induced toxicity.

Mechanistic Pathway	DL-PCB(s)	Model	References
Lipid metabolism			
Epigenetic alterations (↑ miR-155, ↑ miR-34a) → steatohepatitis/fibrosis	PCB-126	C57BL/6 mice	[15,47]
Docosatrienoate depletion as a biomarker of PCB-induced NAFLD	PCB-126 (DL-PCB mixture)	HepaRG cells	[45]
Hepatic fat accumulation (↑ SREBP1c and DGAT-2, ↓ MTP)	PCB-126	Sprague–Dawley strain rats, hepatocytes	[46]
Suppression of PPARα signaling and fatty acids β-oxidation	PCB-126, PCB-169	Mice, hepatocytes	[46,54,59]
Impaired hepatic energy balance (↓ fatty acids β-oxidation)	PCB-126	Male Sprague–Dawley rats, hepatocytes	[59]
Additive toxicity of dioxin-like mixtures (TCDD, PeCDF, PCB-126)	PCB-126 (within DL mixture)	Male and female C57BL/6J mice	[51]
Disruption of adipocyte function and systemic lipid metabolism	PCB-77	Adipocytes, male C57BL/6 mice	[53]
Upregulation of PPARγ/CD36	PCB-169	Male C57BL/6 mice HFD mice	[54]
Increased lipid peroxidation	PCB-77	HepG2, male Sprague–Dawley rats	[54,70,71]
Glucose Metabolism			
Suppressed gluconeogenesis (↓ PEPCK) and ↓ glycogen stores	PCB-126 and other DL-PCBs	Primary mouse hepatocytes, male Sprague–Dawley rats	[55,59]
Interference with HIF-1α hypoxia signaling (competition for ARNT)	PCB-126	HepG2, male Sprague–Dawley rats	[58]
Impaired hepatic energy balance (↓ gluconeogenesis)	PCB-126	Male Sprague–Dawley rats, hepatocytes	[59]
Liver Carcinogenesis			
Induction of pro-tumorigenic processes: EMT via PKM2/STAT3/Snail1 activation (AhR- and ER-dependent)	PCB-126	HCC cells (Bel-7402 and SMMC-7721)	[67]
↑ Neutrophil infiltration and oncogenic liver growth	PCB-126	Kras-transgenic zebrafish	[68]
Iron metabolism			
Induction of lipocalin-2 (LCN2) and modulation by FGF21	PCB-126	HepG2, male C57BL/6 HDF mice	[63]
Iron dysregulation via hepcidin suppression	PCB-126 > PCB-153	Hepatocytes, wild-type and hepcidin-deficient mice	[64]
Oxidative stress and Inflammation			
Upregulation of pro-inflammatory cytokines (TNF-α, IL-1β, IL-6)	PCB-169	Male C57BL/6 mice HFD mice	[54]
GSH/thiol metabolism and oxidation products (4-HNE-GSH, oxylipids) dysregulation	PCB-126	C57BL/6 MCD mice	[3]
Oxidative stress–driven MAPK activation (ERK1/2, JNK, p38)	PCB-126	HepG2 cells	[69]
ROS generation and apoptosis	PCB-77	HepG2, male Sprague–Dawley rats	[70,71]
Other mechanisms			
AhR activation → CYP1A1/1A2/1B1 induction	PCB-126, PCB-77, PCB-81, PCB-169	HepG2, HepaRG, primary hepatocytes, mice, rats	[7,44,60,69]

Abbreviations: Nonalcoholic Fatty Liver Disease (NAFLD), Sterol Regulatory Element-Binding Protein 1c (SREBP1c), Diacylglycerol O-acyltransferase 2 (DGAT-2), Microsomal Triglyceride Transfer Protein (MTP), Peroxisome Proliferator-Activated Receptorα(PPARα), 2,3,7,8-Tetrachlorodibenzo-p-dioxin (TCDD), Pentachlorodibenzofuran (PeCDF), Peroxisome Proliferator-Activated Receptorγ(PPARγ), Cluster of Differentiation 36 (CD36), Phosphoenolpyruvate Carboxykinase (PEPCK), Hypoxia-Inducible Factor 1α (HIF-1α),Aryl Hydrocarbon Receptor Nuclear Translocator (ARNT), Epithelial–Mesenchymal Transition (EMT), Pyruvate Kinase M2 (PKM2), Signal Transducer and Activator of Transcription 3 (STAT3), Snail Family Transcriptional Repressor 1 (Snail1), Aryl Hydrocarbon Receptor (AhR), Endoplasmic Reticulum (ER), Lipocalin-2 (LCN2), Fibroblast Growth Factor 21 (FGF21), High Fat Diet (HFD), Tumor Necrosis Factor α (TNF-α), Interleukin-1β (IL-1β), Interleukin-6 (IL-6), Glutathione (GSH),Glutathione conjugate of 4-hydroxynonenal (4-HNE-GSH), Methionine- and Choline-Deficient (MCD), Mitogen-Activated Protein Kinase (MAPK), Extracellular signal-Regulated Kinases 1 and 2 (ERK1/2), c-Jun N-terminal kinase (JNK), Reactive Oxygen Species (ROS), Cytochrome P450 (CYP). The arrows indicate: → induction; ↑ up-regulation; ↓ down-regulation.

**Table 4:** Key mechanistic pathways involved in NDL-PCBs-induced toxicity.

Mechanistic Pathway	NDL-PCB(s)	Model	References
Lipid and Glucose Metabolism			
Activation of nuclear receptors (NRs) regulating lipid metabolism and xenobiotic responses	Several PCBs	HepG2, in vivo rodent models	[7,73]
Activation of xenobiotic receptors (XRs): PXR and CAR	PCB-153, -180 and other NDL-PCBs	Hepatocytes	[74]
Diet-dependent obesogenic effects →↑ body weight, ↑ adiposity, ↑ hepatic steatosis; Impaired FAs β-oxidation (↓ PPARα, ↓ CPT1A/CPT2) and ↑ lipogenesis (↑ FAS)	PCB-153	Male C57BL/6J HFD mice	[44,91]
Modulation of aquaglyceroporins (AQP3, AQP7, AQP9); Increased lipid accumulation in adipocytes	PCB-101, -153, -180	Mature 3T3-L1 adipocytes	[93]
Indirect CAR activation via PCB–EGFR binding →↓ CAR phosphorylation, ↓ AKT/mTOR	PCB-138, -149, -151, -153, -170, -174, -180, -187	AML-12, HepG2	[35,84]
PI3K/AKT/mTOR pathway impairment →↑ gluconeogenesis, altered energy homeostasis	PCB-151, -153, -170, -180	Hepatic cells	[84]
Reduced hepatic Akt and mTOR phosphorylation	Aroclor 1260	Rodents	[84]
Dysregulation of glucose metabolism →↑ G6PC, ↑ PEPCK, ↓ GSK3β	PCB-153	HepG2	[89]
Insulin secretion alterations and epigenomic remodeling (cAMP pathway, TRP channels, adipocyte lipolysis alteration)	PCB-153	HepG2	[90]
Oxidative Stress, Inflammation and Iron Homeostasis			
Activation of NF-κB) pathway and inflammatory cytokines	PCB-153	AML-12 hepatocytes, 3T3-L1 adipocytes, and C57BL/6 mice	[94]
Oxidative stress: ↓ GPx, ↓ SOD, NF-κB) -mediated apoptosis impairment	PCB-153	Sprague–Dawley rats	[92]
Apoptosis and oxidative stress via TNF-α and Fas signaling	PCB-153, -77	HepG2	[7]
Reduction of redox regulators (HNF1β, GPx1)	PCB-153	AML-12 hepatocytes, 3T3-L1 adipocytes, and C57BL/6 mice	[94]
Disruption of NRF2-mediated antioxidant response	Aroclor 1260	C57BL/6 mice	[56,57]
Severe oxidative stress and systemic toxicity	PCB-153	SXRKO Mice	[95]
Iron overload-mediated oxidative injury in steatosis; Reduction of STAMP2 (iron/copper reductase and redox regulator)	PCB-126, Aroclor1260	C57BL/6 HFD mice	[29]
Disruption of hepcidin–ferroportin axis and estrogen-like repression of hepcidin	PCB-153, -126	HepG2, C57BL/6 mice	[64]
Thyroid Function			
Thyroid hormone (TH) disruption and impaired lipid metabolism	PCB-153, -180, Aroclor 1254, Aroclor 1242	Sprague–Dawley rats	[107]
Competitive binding to transthyretin and increased TH clearance	NDL-PCBs (multiple), hydroxylated/sulfated metabolites	C57BL/6 mice	[104,105,106]
Suppression of thyroid receptor (TR)-mediated transcription	PCB-153, -95, -99, -118, -126	Sprague–Dawley rats	[107]

Abbreviations: Nuclear Receptors (NRs), Xenobiotic Receptors (XRs), Pregnane X Receptor (PXR), Constitutive Androstane Receptor (CAR),Fatty Acids (FAs), Peroxisome Proliferator-Activated Receptor alpha (PPARα), Carnitine Palmitoyltransferase 1A and 2 (CPT1A, 2), Fatty Acid Synthase (FAS), High-Fat Diet (HFD), Aquaglyceroporins (AQPs), Epidermal Growth Factor Receptor (EGFR), Protein Kinase B (AKT), Mammalian Target Of Rapamycin (mTOR), Phosphatidylinositol 3-Kinase (PI3K), Glucose-6-Phosphatase Catalytic Subunit (G6PC), Phosphoenolpyruvate Carboxykinase (PEPCK), Glycogen Synthase Kinase 3 β(GSK3β), Cyclic Adenosine Monophosphate (cAMP), Transient Receptor Potential (TRP), Nuclear Factor Kappa B (NF-κB)), Glutathione Peroxidase (GPx), Superoxide Dismutase (SOD), Tumor Necrosis Factorα (TNF-α), Factor-associated suicide (Fas), Hepatocyte Nuclear Factor 1β(HNF1β), Nuclear Factor Erythroid 2–Related Factor 2 (NRF2), Six Transmembrane Protein Of Prostate 2 (STAMP2), Thyroid Hormone (TH), Thyroid Hormone Receptor (TR).The arrows indicate: → induction; ↑ up-regulation; ↓ down-regulation.

## Data Availability

No new data were created or analyzed in this study.

## Abbreviations

The following abbreviations are used in this manuscript:

Agpat9	Acylglycerol-3-Phosphate O-Acyltransferase 9
AhR	Aryl Hydrocarbon Receptor
ALT	Alanine Aminotransferase
AOPs	Adverse Outcome Pathways
AMPK	Amp-Activated Protein Kinase
AQPs	Aquaglyceroporins
ARNT	Aryl Hydrocarbon Receptor Nuclear Translocator
AST	Aspartate Aminotransferase
ATP	Adenosine Triphosphate
cAMP	Cyclic Adenosine Monophosphate
CAR	Constitutive Androstane Receptor
CCRP	Cytoplasmic CAR Retention Protein
CD36	Cluster of Differentiation 36
CREB	Camp Response Element-Binding Protein
CMRF	Cardiometabolic Risk Factor
CPT1A, 2	Carnitine Palmitoyltransferase 1A and 2
CYP	Cytochrome P450
Dgat1	Diacylglycerol Acyltransferase 1
DGAT-2	Diacylglycerol O-Acyltransferase 2
DL-PCBs	Dioxin-Like PCBs
EGFR	Epidermal Growth Factor Receptor
EMT	Epithelial–Mesenchymal Transition
ER	Endoplasmic Reticulum
ERE	Estrogen Response Element
ERK 1/2	Extracellular Signal-Regulated Kinases 1 and 2
Fabp4	Fatty Acid Binding Protein 4
FAs	Fatty Acids
Fas	Factor-associated suicide
FAS	Fatty Acid Synthase
FGF21	Fibroblast Growth Factor 21
FPN	Hepcidin-Ferroportin
G6P	Glucose-6-phosphate
G6PC	Glucose-6-Phosphatase Catalytic Subunit 1
GGT	Gamma-Glutamyl Transferase
GPx1	Glutathione Peroxidase 1
GSH	Glutathione
GSK3β	Glycogen Synthase Kinase 3 beta
GST	Glutathione S-Transferase
Gyk	Glycerol Kinase
HFD	High-Fat Diet
HGB	Blood Hemoglobin
HIF-1α	Hypoxia-Inducible Factor-1 alpha
HIO	Hepatic Iron Overload
4-HNE-GSH	4-idrossi-2-nonenale-glutathione
HNF1β	Hepatocyte Nuclear Factor 1 beta
Hsp90	Heat Shock Protein 90
ICES-6	International Council For The Exploration Of The Sea
IL-1β	Interleukin-1 beta
IL-6	Interleukin-6
JNK	c-Jun N-Terminal Kinase
LCN-2	Lipocalin-2
MAFLD	Metabolic Dysfunction-Associated Fatty Liver Disease
MAPK	Mitogen-Activated Protein Kinase
MASH	Metabolic Dysfunction-Associated Steatohepatitis
MASLD	Metabolic Dysfunction-Associated Steatotic Liver Disease
MCD	Methionine–Choline Deficient Diet
miRNA	MicroRNA
mtDNA	Mitochondrial DNA
mTOR	Mammalian Target Of Rapamycin
MTP	Microsomal Triglyceride Transfer Protein
NAFLD	Non-Alcoholic Fatty Liver Disease
NASH	Nonalcoholic Steatohepatitis
NDL-PCBs	Non-Dioxin-Like PCBs
NF-κB)	Nuclear Factor Kappa B
NRF2	Nuclear factor erythroid 2-related factor 2
NRs	Nuclear Receptors
OCP	Organochlorine Pesticides
PBREM	Phenobarbital-Responsive Enhancer Module
PCBs	Polychlorinated Biphenyls
PCDDs	Polychlorinated Dibenzo-P-Dioxins
PCDFs	Polychlorinated Dibenzofurans
PeCDF	Pentachlorodibenzofuran
PEPCK	Phosphoenolpyruvate Carboxykinase
PI3K	Phosphatidylinositol 3-Kinase
PKB	Protein Kinase B; AKT
PKM2	Pyruvate Kinase M2
POPs	Persistent Organic Pollutants
PP2A	Protein Phosphatase 2A
PPARα	Peroxisome Proliferator-Activated Receptor Alpha
PPARγ	Peroxisome Proliferator-Activated Receptor Gamma
PXR	Pregnane X Receptor
PXREs	Pregnane X Receptor Response Elements
RARs	Retinoic Acid Receptors
RXRs	Retinoid X Receptors
Snail1	Snail Family Transcriptional Repressor 1
SOD	Superoxide Dismutase
SREBP1c	Sterol Regulatory Element-Binding Protein 1c
STAMP2	Six Transmembrane Protein Of Prostate 2
STAT3	Signal Transducer and Activator of Transcription 3
SXRKO	Steroid And Xenobiotic Receptor Knockout
T3	Triiodothyronine
T4	Thyroxine
TASH	Toxicant-Associated Steatohepatitis
TASLD	Toxicant-Associated Steatotic Liver Disease
TCDD	2,3,7,8-Tetrachlorodibenzo-p-Dioxin
TEF	Toxic Equivalency Factor
TEQ	Toxic Equivalents
THs	Thyroid Hormones
TLR4	Toll-like receptor 4
TNF-α	Tumor Necrosis Factor-alpha
TR	Thyroid Hormone Receptor
TRP	Transient Receptor Potential Channel
UGTs	UDP-Glucuronosyltransferases
VLDL	Very-Low-Density Lipoprotein
XREs	Xenobiotic Response Elements
XRs	Xenobiotic Receptors
